# Silkworm Pupae: A Functional Food with Health Benefits for Humans

**DOI:** 10.3390/foods11111594

**Published:** 2022-05-28

**Authors:** Yaxi Zhou, Shiqi Zhou, Hao Duan, Jing Wang, Wenjie Yan

**Affiliations:** 1 College of Biochemical Engineering, Beijing Union University, No. 18, Caili District 3, Futou, Beijing 100023, China; 15239407080@163.com (Y.Z.); shiqizhougood@163.com (S.Z.); dhuanao@163.com (H.D.); 2 Beijing Key Laboratory of Bioactive Substances and Functional Food, College of Biochemical Engineering, Beijing Union University, 197 North Tucheng West Road, Beijing 100023, China; 3 Institute of Food and Nutrition Development, Ministry of Agriculture and Rural Affairs, No. 12 Zhongguancun South Street, Beijing 100081, China; wangjing07@caas.cn

**Keywords:** silkworm pupae, composition, functions, applications

## Abstract

Silkworm pupae are insects that are beneficial to human health, not only for their high nutritional value but, more importantly, for the variety of pharmacological functions they can perform when consumed. Currently, there is a lot of interest in the pharmaceutical applications of silkworm pupae. In recent years, the biological functions of domestic silkworm pupae have gradually been identified and confirmed, especially for their beneficial effects on human health. Studies have found that silkworm pupae have positive effects on liver protection, immune enhancement, antiapoptosis, antitumour, antibacterial, regulation of blood glucose and blood lipids, and lowering of blood pressure. However, the pharmacological mechanisms and systemic safety of silkworm pupae have not been systematically evaluated. In this paper, the nutritional composition of the pupae of the domestic silkworm is first summarised. The pharmacological functions of silkworm pupae and their components are then classified, and their mechanisms of occurrence are described. In addition, we provide a preliminary evaluation of the safety of silkworm pupae, analyse their application prospects, and suggest future directions for further pharmacological function studies. The aim is to generate interest in the promotion of human health through the use of silkworm pupae.

## 1. Introduction

**Figure 1:**
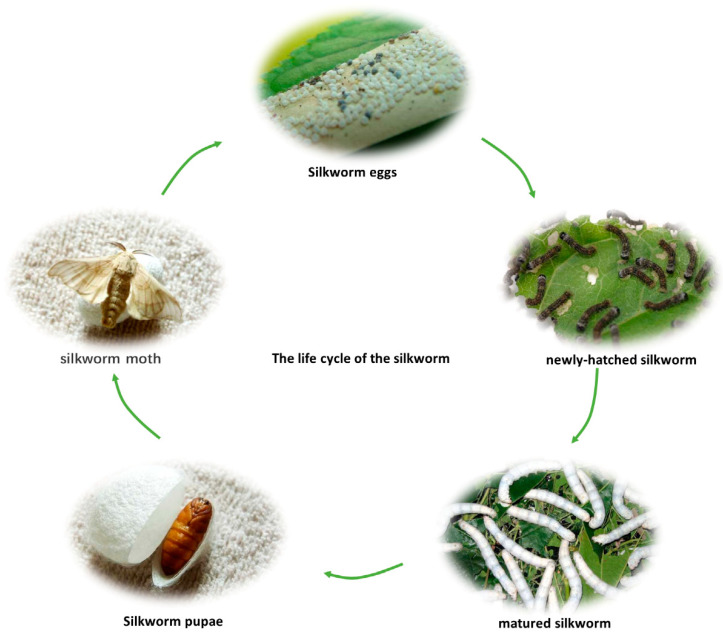
The life cycle of the silkworm.

The silkworm is a lepidopteran insect. The life of a silkworm usually goes through five stages, lasting a total of about seven weeks. When the silkworm eggs hatch, they turn into newly hatched black and brown silkworms. After feeding and growing and shedding its shell five times, the silkworm becomes a mature silkworm, stops feeding, and starts to spit out a lot of silk in preparation for cocooning. This process takes 24–28 days. After 4 days of cocooning, the matured silkworm turns into a silkworm pupa. After about 2 weeks, the silkworm pupae turns into silkworm moths. The silkworm moths finish laying eggs within 3–5 days and die soon afterwards. Silkworm pupae are considered to be the harvest period for silkworm consumption as they are consumed as food in many regions due to their high nutritional value and various biomedical functions. Figure 1 depicts the life cycle of the silkworm [1,2].

Silkworm pupae are one of the main by-products of the silk industry and are mostly used as animal feed and fertiliser in South East Asia, for example in Japan, Korea, and India [3,4,5]. Silkworm pupae are also used as food insects, especially in China, where they have been eaten for over 2000 years [6]. There are many species of silkworm pupae; at present, the main commercial silkworm pupae used for research are *Bombyx mori*, *Antheraea pernyi*, *Antheraea yamamai*, *Samia ricini*, *Antheraea mylitta*, *Antheraea roylei*, and other species [4,7,8]. Additionally, the material composition and functional roles of these species are different. The biggest difference between the different species of silkworm pupae is the source of the silkworm’s diet and the degree of domestication. For example, the mulberry silkworm, which eats mulberry leaves, is the silkworm that has been fully domesticated and is the most widely farmed [4]. Rich in proteins, oils, chitosan, vitamins, polyphenols, and other nutrients, silkworm pupae have long been used as an important source of high-quality proteins and lipids [9]. Silkworm pupae protein contains 18 amino acids and is rich enough in essential amino acids to meet the amino acid requirements of humans and is beneficial to human health [10,11]. Silkworm pupae oil contains a large number of unsaturated amino acids, especially Omega-3 fatty acids [12].

Silkworm pupae have long been used in a single way for direct consumption, for example as feed [5]. Gradually, the pupae have been further processed to extract nutrients and active ingredients and are used in food modification and pharmaceutical development [13]. Some researchers have used silkworm pupa powder as a protein enhancer in functional foods, and the addition of silkworm pupa powder enhances the flavour and taste of functional foods [13,14,15]. Examples are bread, yogurt, and food additives [7,13,16]. In addition, silkworm pupae can also be used in industry [17]. However, silkworm pupae are still not accepted by everyone because of the presence of allergens and unfriendly odours. We need to consider the safety and acceptability of silkworm pupae more thoroughly when using them [18]. In recent years, numerous studies have found that the active ingredients in silkworm pupae have various pharmacological functions, such as: anticancer, antioxidant, hepatoprotective, antibacterial, antiapoptotic, and immunomodulatory functions. This provides a broader prospect for the application of silkworm pupae. In the future, silkworm pupae will rapidly be developed for the health food and biomedical industries to meet the human demand for nutritious food and safe medicine [19,20].

This article reviews information on silkworm pupae as food and a medical ingredient. It covers data from biochemistry, nutrition, biomedicine, and pharmacology. The text discusses the composition and functional mechanisms of action of silkworm pupae and analyses safety issues and application prospects for their use, with the aim of revealing the link between silkworm pupae and health and providing a reference for their pharmacological application.

## 2. Components of Silkworm Pupae

Silkworm pupae are rich in many nutrients. Protein, fat, and sugar are the most abundant substances, as well as minerals, vitamins, polyphenolic compounds, and many other nutrients [21,22]. Here, we have discussed and summarised them separately.

### 2.1. Silkworm Pupae Protein

*Bombyx mori* has a high protein content of 55.6% dry weight and is the most abundant dry matter in silkworm pupae [9].Biologically active peptides are peptides containing from several to several dozen amino acids, which have a variety of physiological functions [23]. These pupae proteins can be hydrolysed to produce a variety of biologically active peptides, which in turn can perform the pharmacological functions of silkworm pupae. The amino acid composition of the proteins is essentially the same in the different species of silkworm pupae, all consisting of 18 amino acids (except for *Eri* silkworm pupae). Of these, eight essential amino acids meet the requirements of the WHO/FAO/UNU recommendations. In addition, there are 10 non-essential amino acids that meet human requirements. Compared to hen eggs, pupae are higher in Phe and Pro [24]. Therefore, silkworm pupae are considered to be a high-quality source of protein and an important nutrient in silkworm pupae [25]. Table 1 summarises the amino acid composition of the different varieties of silkworm pupae proteins.

### 2.2. Silkworm Pupae Oil

In silkworm pupae, the oil content is second only to protein. Of the four different species of silkworm pupae, *Eri* silkworm pupae has the highest oil content, at 26.2% [22]. We summarise the fatty acid composition of the different varieties of silkworm pupae oil in Table 2. As can be seen from the table, all the different silkworm pupae oils contain high levels of unsaturated fatty acids, with 77.71% in *Antheraea pernyi*. In addition to the fatty acids listed in the table, silkworm pupae also contain eicosapentaenoic acid and docosahexaenoic acid, which are Omega-3 fatty acids and have an important role in promoting human health [26]. Oil is an important nutrient, and silkworm pupae are not only rich in oils but also contain high levels of unsaturated fatty acids, especially polyunsaturated fatty acids, which have significant nutritional value as a source of edible oil [12].

### 2.3. Minerals

Minerals have an important role in living organisms. They are present in many forms in silkworm pupae. There are up to 25 different types of minerals in silkworm pupae, and these minerals may perform certain physiological functions in the organism [7,12]. Table 3 lists the content of eight minerals in the three types of pupae, from which it can be seen that phosphorus, calcium, and magnesium are higher in the pupae. The type and content of minerals in pupae can vary depending on the type of pupa and the environment in which they have grown [24]. It is worth noting that the sodium-to-potassium (Na: K) ratio in silkworm pupae is very low, except for the minerals listed in the table. Na:K predicts the occurrence of non-communicable diseases, suggesting that consumption of silkworm pupae may reduce the likelihood of non-communicable diseases [29,30]. Non-communicable diseases include stroke, hypertension, cardiovascular disease, etc. [31,32]. Some pupae are also rich in selenium, which can be enriched in the pupae protein. Selenium-rich pupae play an important role in cancer prevention and defence against oxidative stress [33,34].

### 2.4. Other Ingredients in Silkworm Pupae

In addition to the above ingredients, silkworm pupae contain many vitamins and are rich in them. For example, VA can reach 5 mg/g. The main vitamins in silkworm pupae include VA, VB1, VB2, VB3, VB5, VB7, VB9, VB12, VC, and VE [19,36]. Phospholipids and five tocopherols are also present in silkworm pupae. The five tocopherols are α-Tocopherol, β-tocopherol, γ-tocopherol, γ-tocotrienol, and σ-tocopherol [28]. Rare dimethyladenosine derivatives are also found in silkworm pupae [37]. Additionally, silkworm pupae contain polyphenols and flavonoids. Polyphenols and flavonoids were found in the pupae of the silkworm *Antheraea assamensis* at concentrations of 10 mg/g and 20 mg/g, respectively [35]. In native *Thai* mulberry silkworm pupae, the polyphenols mainly contain (+)-catechin, (−)-epicatechin, rutin, quercetin, myricetin, trans-resveratrol, luteolin, naringenin, and kaempferol [38]. The sugars in silkworm pupae can be divided into two main groups, chitosan and chitin, as well as isolated and purified polysaccharides, all of which are biologically active [39,40,41]. Chitosan and chitin from silkworm pupae are not cytotoxic, but have strong physiological activity, especially carboxymethyl chitosan [42,43]. All these substances had certain biofunctional activities, which contribute to the basis of the pharmacological functions of silkworm pupae.

## 3. Pharmacological Functions and Mechanisms of Silkworm Pupae

The active ingredients in silkworm pupae have a variety of pharmacological functions and have significant therapeutic effects on many diseases. Both in vivo and in vitro experiments have shown the powerful pharmacological effects of silkworm pupae. Examples of these effects include antitumor, antioxidant, antibacterial, antiapoptotic, hypotensive, lipid- and blood-sugar-regulating, immunomodulatory, and hepatoprotective effects. The pharmacological functions of silkworm pupae are summarised below, and detailed information is given in Table 4.

### 3.1. Anticancer Effect

**Figure 2:**
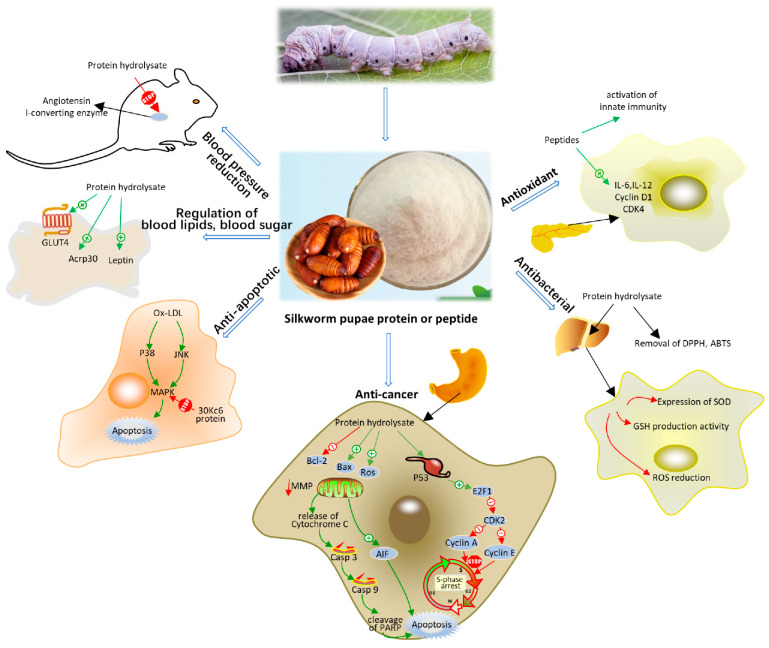
Function and physiological mechanisms of silkworm pupae proteins.

Currently, cancer is mostly treated with radiotherapy and chemotherapy, which have side effects on the body, so finding natural antitumor drugs would be a better option. In vitro studies found the protein hydrolysates and amino acids in silkworm pupae had anticancer effects and were cytotoxic to human stomach cancer cells, breast cancer cells, and liver cancer cells [33,44,46]. Both silkworm pupa protein and silkworm pupa oil were found to have anticancer activity. Silkworm pupae proteins act as anticancer agents by affecting the division cycle of cancer cells and inducing the production of apoptotic factors to promote apoptosis. In Figure 2, the mechanism of the anticancer effect of silkworm pupae protein is shown in detail. In addition, silkworm pupae protein also affects the mitochondria of cancer cells, which in turn affects the energy metabolism function of cancer cells and activates the apoptotic flux, causing cancer cells to die [45]. Unlike the anticancer effects of silkworm pupae proteins, the mechanism of action of amino acids in silkworm pupae oil to inhibit liver cancer is to induce apoptosis through the production of ROS in cancer cells [33]. If further research continues, silkworm pupae might be useful as a raw material for antitumor drugs in the treatment of cancer. 

### 3.2. Antioxidant Activity

Nowadays, a variety of peptides and polyphenols with antioxidant effects are isolated and extracted from silkworm pupae by different methods, and in vitro experiments have shown that these substances are active in scavenging DPPH and ABTS free radicals and scavenging intracellularly generated ROS [47,48,50]. A study found that two peptides extracted from silkworm pupae exhibited strong antioxidant activity in HepG2 cells, as evidenced by ROS reduction, superoxide dismutase (SOD) expression, and glutathione (GSH) production activity [48]. In DPPH and ABTS radical scavenging assays, a 30% ethanolic extract of silkworm pupae was found to have the highest antioxidant activity. Furthermore, the activity of antioxidants in pupae was found to differ by sex and age, with stronger free radical scavenging and ROS scavenging in female pupae in early pupation [50]. The activity of antioxidants can vary depending on the method of extraction. A study on the extraction of silkworm pupa oil by microwave-assisted extraction found that the use of microwave-assisted extraction not only increased the yield of silkworm pupa oil but also resulted in a higher total phenolic content in the oil, which in turn led to stronger antioxidant activity than that achieved with the normal extraction method [27]. The unsaturated fatty acids, peptides, and phenolic compounds in silkworm pupae exhibit antioxidant activity, and it might be possible to develop foods or medicines with antioxidant properties using silkworm pupae. Because of their hydrophilicity, permeability, and multifactorial interaction with the biological environment, natural antioxidants play an important role in disease prevention. Synthetic antioxidants, on the other hand, may interfere with other nutrients’ biological functions in the body. As a result, natural antioxidants are frequently seen as being safer than synthetic antioxidants [77,78]. Because these substances are natural antioxidants, the effect might be better and safer.

### 3.3. Antibacterial Activity

Although silkworm pupae have a long history of use in medicine, research into their antibacterial effects has only recently become more advanced. Firstly, it was found that silkworm pupa oil has antibacterial activity. The antimicrobial activity of silkworm pupa oil was determined using the minimum inhibitory concentration (MIC) method, and it was found that it significantly inhibited the growth of a Staphylococcus sciuri strain CD97, with the best effect being achieved at 110 µL/mL [79].In addition, the antibacterial activity of hot-pressed extracted silkworm pupa oil was also found to be more pronounced in Gram-positive bacteria [51]. Silkworm pupae shells are rich in chitin and chitosan, which have good antibacterial properties and have been used in various biomedical applications [80,81]. It was found that the chitosan in silkworm pupae was 48% crystalline and 67% acetylated. The antibacterial and antifungal activity of chitosan from silkworm pupae was better than that of commercially available chitosan, with the fastest inhibition of bacteria being achieved at 1–2 h [40]. The antibacterial component of silkworm pupae could be used as a resource for treating diseases and could be used to reduce the high use of antibiotics.

### 3.4. Antiapoptotic Effect

Silkworm pupae are rich in a low-molecular-weight lipoprotein that has been shown to be a member of the 30 K family of proteins that transport lipids and inhibited apoptosis in mammalian cells [53,54,82]. Initially, researchers found that the haemolymph of silkworm pupae had apoptosis-inhibiting activity against virus-infected insect cells and that the addition of silkworm pupae haemolymph increased the lifespan of virus-infected cells. Subsequently, a non-glycosylated monomeric protein with antiapoptotic activity was purified from the haemolymph [53]. Later, it was confirmed that the substance exerting the antiapoptotic function was a 30 K protein, and a recombinant 30 K protein was expressed in *E. coli*, which, like haemolymph, inhibited viral or chemically induced apoptosis [54]. Further studies revealed that silkworm pupae haemolymph also inhibited the onset of apoptosis in human cells [55]. These findings are of great benefit to cell culture in vitro.

### 3.5. Regulation of Blood Pressure, Blood Sugar, and Blood Lipids

In hypertensive patients, angiotensin-converting enzyme activity is enhanced, while silkworm pupae protein hydrolysate significantly inhibits angiotensin-converting enzyme activity. The inhibition activity of the angiotensin-converting enzyme was 73.5% at a concentration of 2.0 mg/mL of silkworm pupa protein hydrolysate, while the optimised hydrolysis method resulted in a semi-inhibitory concentration of 1.4 mg/mL [57]. In addition, a peptide isolated from silkworm pupae protein had a strong inhibitory effect on angiotensin-converting enzyme activity, with a semi-inhibitory concentration of 0.047 mg/mL [59]. After four weeks of feeding different concentrations of pupa protein hydrolysate to spontaneously hypertensive rats, it was found that the systolic blood pressure of the rats decreased, and the decrease was dose-dependent on the concentration of the hydrolysate [56]. Silkworm pupae protein hydrolysate have shown hypotensive activity in vitro and in vivo, and therefore could be developed and applied as an antihypertensive drug or an antihypertensive food supplement. Silkworm pupae also have a regulating effect on blood sugar and blood lipids. Silkworm pupae powder acts as an alpha-glucosidase inhibitor and lowers postprandial blood sugar levels. It also promotes fat metabolism and reduces fat accumulation in rats [61,62,64]. This means that silkworm pupae have the potential to be developed as a drug to lower blood sugar levels in diabetics with the same weight loss effect.

### 3.6. Gastric and Hepatoprotective Effects

Animal studies have found that silkworm pupae oil has a protective effect against hydrochloric-acid/ethanol-induced gastric ulcers. Silkworm pupae oil reduced the area of gastric ulcers and gastric secretions and increased the PH in the stomach of mice. In mice with gastric ulcers, silkworm pupae oil increased serum levels of SOD, CAT, GSH-Px, SST, VIP, and decreased levels of IL-6, IL-12, TNF-α, IFN-γ, MTL, GT. Meanwhile, the expression of EGF, EGFR, VEGF, and eNOS was promoted, and the expression of NF-κB, Bcl-2, COX-2, and iNOS was reduced. This evidence indicates that the use of silkworm pupae oil reduces oxidative damage and inflammatory responses in mice [69]. Silkworm pupae oil also reduced acetaminophen-induced acute liver injury and alcohol-induced hepatotoxicity and oxidative stress in mice by inhibiting the oxidative-stress-mediated NF-κB signalling pathway [67,68]. Silkworm pupae might serve as a potential drug resource for the treatment of gastric ulcers and the prevention of acute liver injury.

### 3.7. Cardiovascular Protection

Not only does the 30 K protein in silkworm pupae possess antiapoptotic activity, but studies have also found that the 30 K protein has protective effects against cardiovascular disease. In an atherosclerotic rabbit model, silkworm pupae 30Kc6 protein reduced serum levels of total triglycerides (TG), high-density lipoprotein cholesterol (HDL-C), low-density lipoprotein cholesterol (LDLC), and total cholesterol (TC), and reduced the extent of aortic and liver lesions in atherosclerotic rabbits [52]. Crude extracts of silkworm pupae have also been reported to improve the condition of atherosclerotic rabbits, presumably through antioxidant and hypolipidemic effects [65]. Silkworm pupae oil sodium salt was found to significantly reduce platelet-derived growth-factor-induced abnormal migration and proliferation of vascular smooth muscle cells. Silkworm pupae oil sodium salt treatment down-regulates ERK1/2 phosphorylation levels in vascular smooth muscle cells [66]. The findings of these studies could inform the prevention and treatment of cardiovascular disease, perhaps via the development of medicines or functional foods for the treatment of cardiovascular disease.

### 3.8. Immunomodulatory Effects

Silkworm pupae are high-protein content insects. Several novel immunomodulatory peptides have been isolated and purified from silkworm pupae, and these active peptides could play an important role in the regulation of immunity [83]. It has been reported that researchers have purified a novel immunomodulatory peptide with a molecular weight of 441.06 Da and an amino acid sequence of Asp-His-Ala-Val from silkworm pupae protein hydrolysate. This immunomodulatory peptide stimulated the expression of IL-6, IL-12, NF-κB, Cyclin D1, and cyclin-dependent kinase 4 [70]. Insects are an important source of active peptides, and their immunological effects on aquatic animals have been comprehensively reported, with insect consumption improving the immune status of aquatic animals [84]. This means that it is feasible to search for immunomodulatory substances in insects. An immunologically active polysaccharide has also been identified in the pupae, which activated the innate immunity of RAW264 cells and small shrimps, thereby increasing their survival rate [41]. Through intensive research, silkworm pupae proteins and polysaccharides with immunomodulatory activity might have therapeutic effects on diseases related to the human immune system.

### 3.9. Anti-Alzheimer’s Disease

Silkworm pupae have ameliorating effects on many neurological disorders associated with oxidative stress, for example, stroke and Alzheimer’s [85]. In vivo studies in animal models of Alzheimer’s disease have shown that silkworm pupae can enhance cognitive function in Wistar rats by increasing cholinergic function and exerting neuroprotective effects by reducing oxidative stress. Study results demonstrate that silkworm pupae reduce memory impairment and hippocampal neuronal density in an animal model of Alzheimer’s disease [74].In addition, researchers have developed an Alzheimer’s disease vaccine using silkworm pupae. Transgenic mice for Alzheimer’s disease receiving recombinant proteins expressed in silkworm pupae showed reduced brain deposition, lower malondialdehyde levels, and improved memory and cognitive performance [73]. Silkworm pupae could be a potential functional food for the prevention of Alzheimer’s disease and might be able to provide a therapeutic pathway for Alzheimer’s disease in humans. Again, this demonstrates the potential value of silkworm pupae in vaccine development and provides a reference for the development of new vaccines using silkworm pupae.

### 3.10. Other Important Pharmacological Functions of Silkworm Pupae

**Figure 3:**
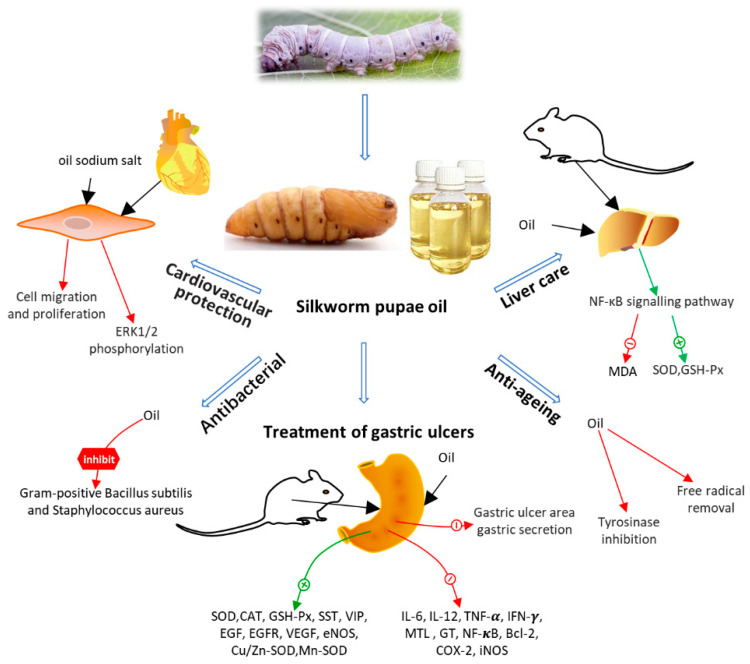
Function and physiological mechanisms of silkworm pupae oils.

In addition to the above pharmacological functions, silkworm pupae also have antifatigue [72,86], antiaging [76], antigenotoxic [35], and alcohol detoxifying [75] effects and inhibit the proliferation of fibroblasts [43]. Moreover, silkworm pupae are suitable as bioreactors for the expression of heterologous proteins, which is important for the development of vaccines and the production of recombinant proteins [71]. Here, we summarise the functional mechanisms of action of silkworm pupa protein (Figure 2) and silkworm pupa oil (Figure 3), respectively.

## 4. Safety Evaluation of Silkworm Pupae

Silkworm pupae contain a wide range of bioactive components, and these have a variety of pharmacological functions. However, when we consider whether pupae can be food and medicine, the safety of their use must be assessed. Safety is the most basic human need for any food or medicine. Silkworm pupae are generally safe as food and safer than other common high-protein foods such as seafood and fish [6,87]. However, studies and safety evaluations are still needed. In general, safety concerns for silkworm pupae are expressed in terms of both toxicological safety and allergic reactions.

For transgenic silkworms, researchers conducted a 28-day feeding study on rats, which was used to assess subacute toxicity. The study found no adverse reactions or deaths in rats fed the transgenic silkworms, indicating that the transgenic silkworms were toxicologically safe, at least for consumption by rats [88]. The investigators also evaluated the safety of silkworm pupae protein using a series of acute and subacute toxicological tests (acute toxicity test; teratogenicity test; and 30-day feeding study). The results show that no death or abnormal haematological, clinical chemical, or histopathological changes were observed in any of the experimental groups. The maximum dose of silkworm pupae protein in rats is 1.50 g/kg/d, which is generally considered safe [89]. Although the oil is high in unsaturated fatty acids and of good quality, it is not recommended for direct consumption due to the presence of triacylglycerols. A study found that structured triacylglycerols synthesised using solvent-free systems are more suitable for direct human consumption. This provided an approach to the safe use of silkworm pupae oil [90].

Allergy to silkworm pupae is now widely studied. The allergenicity of silkworm pupae has been extensively reported, and the key allergen is the silkworm pupae protein or peptide, such as 27-kDa glycoprotein, Chitinase precursor, Paramyosin, and Profilin [18,91,92,93]. Twenty-six protein-based allergens which can cause hives, dizziness, itchy skin, and even shock in some people who consume silkworm pupae have been identified [6,91,94]. Allergic reactions can limit the use of silkworm pupae in food or health products, so it is important to find ways in which allergens can be reduced. Currently, many feasible methods or operations have been explored to reduce or mitigate the allergenicity of silkworm pupae.

A study found lower allergen levels in female silkworm pupae reared on mulberry leaves, suggesting that the production of silkworm pupae allergens may be related to gender and diet [95]. Although we cannot completely avoid the production of these allergens, we can regulate them in the production or use of silkworm pupae. In addition to this, when processed as food, heat causes conformational changes and chemical modifications to the allergen, resulting in the loss or reduction in allergenicity [96,97]. In the food industry, hydrolysis and fermentation can satisfactorily reduce the allergenicity of foodstuffs, so hydrolysis and fermentation can be considered for the pretreatment of silkworm pupae [98]. A recent study found that heat, enzymatic digestion, and acid–base treatment significantly reduced the allergenicity of silkworm pupae protein extracts. It can be assumed that high pressure, ultrasound, and microwave treatments also have an effect on the allergens in the pupae [99].

Overall, silkworm pupae are safe as food or medicine. Although allergens in silkworm pupae can trigger allergic reactions, they can be destroyed and reduced in a variety of ways.

## 5. Application Prospects of Silkworm Pupae

Due to their properties, silkworm pupae have been widely used in food, pharmaceutical, textile, and other industries [22,100,101]. Silkworm pupae are being used to develop new food products, and pupae protein and oil have been reported as alternative sources of protein and fat for bread making [13].In the future, the consumption of silkworm pupae will not just be limited to direct consumption, but a variety of new food products will be developed that contain silkworm pupae. As pupae are rich in protein, they can be used as a high-protein food for human consumption and as an ideal material for nutritional feed for animals [4,22].In addition, the high-protein properties of silkworm pupae can be used as bioreactors to express exogenous proteins [71]. The development of recombinant protein and virus vaccines using silkworm pupae is not only easy to perform but also safe, stable, and cost-effective. Several vaccines have been successfully expressed in vivo or intracellularly in silkworm pupae, e.g., the influenza vaccine [102,103] and HPV vaccine [104]. A recombinant protein developed using silkworm pupae could improve memory and cognitive performance in mice, which would have potential applications in the prevention of Alzheimer’s disease [105]. Similarly, the high quality of chitosan and chitin in silkworm pupae indicates their potential in biomedicine as pharmaceutical preparations or drug carriers [106].

In the future, the medicinal use of silkworm pupae will be a hot topic of research, and people are becoming aware that they can be used for more than just food [100]. From the evaluation of the results of the studies summarised in Table 4, it is predicated that silkworm pupae may be developed for a variety of medicinal products in the future. Examples include anticancer drugs, antioxidants, blood-sugar- and lipid-lowering drugs, antihypertensive drugs, drugs for cardiovascular diseases, anti-inflammatory drugs, or immune-boosting supplements. As a potential medicinal insect, the development of medicinal functional products from silkworm pupae is of great importance to human health. Silkworm pupae are widely used in biomedicine and will be an important biological resource [20,107].

## 6. Laws and Regulations on Silkworm Pupae as Edible Insects

The US Food and Drug Administration (FDA) is very concerned about the use of insects as human food, as evidenced by FDA (2016) regulations. According to the FDA (2016), insects should be used as a food source by humans. The premise of pupae as edible insects is backed up by relevant laws and regulations, which require a joint effort from legislators, regulators, and law enforcement [108]. New EU food legislation allows for the legalisation of edible insect consumption in Europe [109]. However, safety of both the producer and the consumer is something that must be considered [110]. For the development and use of silkworm pupae, more comprehensive and clear regulations are needed to govern the production and consumption of insect food products similar to silkworm pupae. This could be used not only to promote the relationship between silkworm pupae and human health, but also as a form of protection for consumers [111].

## 7. Conclusions

This review summarises the composition of silkworm pupae, highlights the mechanisms of functional occurrence of silkworm pupae as a functional food and medicine, and illustrates the potential of silkworm pupae as a dietary supplement and medicine for the treatment of disease. The main advantages of silkworm pupae are that they are particularly nutritious and can be produced in large quantities in a short time. They also have a wide range of bioactive functions. This makes silkworm pupae promising for use in the biomedical and pharmaceutical fields. However, the current research on the pharmacological mechanism of action of silkworm pupae is not thorough enough, and many studies on the pharmacological functions only remain at the level of in vitro experiments and animal experiments. Researchers urgently need to conduct clinical trials to validate the pharmacological functions of silkworm pupae. In addition, there is not a wide range of healthy food and pharmaceutical products developed from silkworm pupae, and the use of silkworm pupae by humans is still in its infancy. The results we have summarised show that silkworm pupae have great potential for biomedical applications. In the future, researchers should focus on exploring the pharmacological functions of silkworm pupae, both at the molecular level and in clinical trials, so that they can be used to promote human health.

## Figures and Tables

**Table 1:** Amino acid composition of different varieties of silkworm pupae proteins [9,21,22,24].

Amino Acid (g/100 g of Protein)	*Bombyx mori*	*Eri* Silkworm Pupae	Mulberry Silkworm Pupae	*Antheraea pernyi*	Hen Egg
Asp	9.1	9.89	10.9	6.41	8.92
Thr	3.9	4.75	5.4	4.64	4.47
Ser	3.7	5.25	4.7	4.64	6.72
Glu	9.5	12.9	14.9	12.74	12.13
Gly	3.6	4.94	4.6	4.42	3.02
Ala	3.9	6.05	5.5	6.26	5.03
Cys	1.4	0.53	1.4	1.5	1.90
Val	4.7	5.36	5.6	6.63	5.42
Met	3.4	2.31	4.6	1.47	2.81
Ile	3.4	4.42	5.7	7.95	4.88
Leu	6.2	6.63	8.3	3.24	8.11
Tyr	5.6	6.4	5.4	2.06	3.81
Phe	4.6	5.24	5.1	8.10	4.82
Lys	6.1	6.54	7.5	4.54	6.59
His	2.7	2.67	2.5	2.94	2.09
Arg	4.7	4.41	6.8	4.12	5.70
Pro	7.0	6.46	4.0	12.22	3.38
Trp	1.5	NA	0.9	4.05	1.72

Values are expressed as g/100 g of protein. NA: Data not available.

**Table 2:** Fatty acid composition of different varieties of silkworm pupae oil [9,12,19,26,27,28].

Fatty Acids (Percentage of Fatty Acids)	Chemical Structure	*Bombyx mori*	*Eri* Silkworm Pupae	Mulberry Silkworm Pupae	*Antheraea pernyi*	Sunflower Oil
Myristic acid (C14:0)	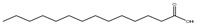	0.1	ND	0.18	NA	NA
Palmitic acid (C16:0)	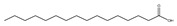	24.2	26.98	23.18	17.25	5.6
Palmitoleic acid (C16:1)	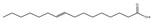	1.7	1.82	1.07	1.14	NA
Stearic acid (C18:0)	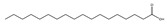	4.5	4.73	4.69	2.23	2.2
Oleic acid (C18:1)	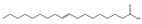	26.0	15.89	28.32	29.15	25.1
Linoleic acid (C18:2)	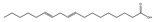	7.3	5.49	3.88	7.14	66.2
α-Linolenic acid (C18:3)	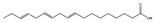	36.3	44.73	38.25	40.28	NA
Saturated fatty acids	—	28.8	31.71	28.05	19.48	7.8
Monounsaturated fatty acids	—	27.7	17.71	29.39	30.29	25.1
Polyunsaturated fatty acids	—	43.6	50.22	42.13	47.42	66.2

Values are expressed as a percentage of fatty acids. NA: data not available.

**Table 3:** Mineral composition of different species of silkworm pupae [21,22,24,35].

Minerals (mg/100 g Dry Weight)	*Bombyx mori*	*Eri* Silkworm Pupae	*Antheraea pernyi*
Phosphorus	474	584	272
Iron	26	24	4
Calcium	158	74.2	63
Zinc	23	7.24	3.57
Copper	0.15	1.75	0.73
Magnesium	207	178	154
Manganese	0.71	2.54	NA
Chromium	1.69	NA	9.84

Values are expressed as mg/100 g dry weight. NA: data not available.

**Table 4:** Pharmacological functions and mechanisms of occurrence of silkworm pupae.

Pharmacological Functions	Species of Silkworm Pupae	Functional Ingredients	Cell Line/Animal Model or Method	In Vivo or In Vitro	Occurring Mechanism or Effect	Evaluation of Research Findings	Reference
Antitumor	*Bombyx mori*	Protein hydrolysates	Human gastric cancer SGC-7901 cells	In vitro	Inhibits the proliferation of human gastric cancer cells SGC-7901 and stimulates their abnormal morphological features; induces apoptosis and blocks the cell cycle in S phase; causes the accumulation of ROS and depolarisation of the mitochondrial membrane potential.	Silkworm pupae could be a source of anticancer drugs in the future.	[44]
	*Bombyx mori*	Protein hydrolysates	MGC-803 gastric cancer cells	In vitro	Structural changes in intracellular organelles, including mitochondrial swelling, vacuolisation and rupture. Impact on the metabolic energy supply of MGC-803 cells.	Represents a potential chemotherapy candidate for the treatment of gastric tumours.	[45]
	*Bombyx mori*; *Samia ricini*	Protein extracts	Breast cancer cells MCF-7	In vitro	MCF-7 cells had significantly lower protein and nucleic acid content, as well as significantly lower IL-6, IL-1, and TNF- levels.	May provide a potential novel therapeutic target for breast cancer.	[46]
	*Ziyang silkworm pupae*	Se-rich amino acids	Human hepatoma cells	In vitro	Significantly and dose-dependently inhibits cell viability, induces changes in cell morphology and cycle, and causes apoptosis. It induces apoptosis through the production of ROS.	It could be used as an anticancer medicine or as a food source of necessary amino acids and trace elements for everyday health.	[33]
Antioxidant	*Antheraea assamensis*	Methanolic pupae extract	—	In vitro	Free-radical scavenging activity that is dose-dependant.	—	[35]
	*Bombyx mori*	Protein hydrolysates	—	In vitro	ABTS free-radical scavenging activity is strong.	Potential usage as a natural antioxidant in functional foods to help prevent diseases caused by oxidative stress.	[47]
	*Bombyx mori*	Protein hydrolysates	Hepatic HepG2 cells	In vitro	In HepG2 cells, the antioxidant activity was highest (ROS reduction, superoxide dismutase expression, and glutathione synthesis activity).	May have potential as natural antioxidants.	[48]
	*Bombyx mori*; *Antheraea mylitta*; *Antheraea assamensis*	Polyphenols	—	In vitro	High ROS scavenging activity was observed.	Effective as a natural antioxidant in the development of protein-rich foods.	[49]
	—	30% ethanol extract	HepG2 cells	In vitro	Scavenging action against DPPH and ABTS; helping remove ROS.	Silkworm pupae can be used to generate culinary ingredients and functional materials.	[50]
Antibacterial	*Bombyx mori*	Chitin and chitosan	*Bacillus cereus*; *Staphylococcus aureus*; *E. coli*; *Klebsiella pneumonia*	In vitro	Antifungal activity is comparable to, if not superior to, commercially available chitosan. Bacterial inhibition was greatest between 1 and 2 h and began to achieve saturation after 24 h.	Silkworm pupae are a renewable and sustainable source of chitosan that may be used in both food and medicine.	[40]
	*Bombyx mori*	Peptides	—	In vitro	The Peptide Ranker and the CAMP (Collection of Anti-Microbial Peptides) database found peptide sequences with potential bioactivity with the highest score.	It can be utilised as a high-quality protein source.	[25]
	*Bombyx mori*	Oil	*Pseudomonas aeruginosa* PAO1; *Escherichia coli* C1a; *Staphylococcus aureus* ATCC 6538P; *Bacillus subtilis* ATCC 6633	In vitro	Gram-positive *Bacillus subtilis* and *Staphylococcus aureus* were sensitive to *H. illucens*- and *B. mori*-derived oils, but Gram-negative *Pseudomonas aeruginosa* and *Escherichia coli* were not.	Silkworm pupae oil can be used as an effective antibacterial agent.	[51]
Antiapoptotic	*Bombyx mori*	Silkworm Protein 30Kc6	The in vitro cell apoptosis model of HUVEC that was induced by oxidised low-density lipoprotein.	In vitro	Cell-mitogen-activated protein kinases (MAPK), particularly JNK and p38, were activated by oxidised low-density lipoprotein; 30Kc6 prevented oxidised low-density lipoprotein-induced cell death in HUVEC cells by blocking MAPK signalling pathways.	It has the potential to provide crucial information for human cardiovascular disease prevention and treatment.	[52]
	*Bombyx mori*	Silkworm haemolymph	Insect cells (Sf 9) infected with baculovirus (AcNPV)	In vitro	The silkworm’s haemolymph may directly affect the baculovirus-induced apoptosis cascade or promote the expression of antiapoptotic baculovirus genes such as p35.	Antiapoptotic components can be found in silkworm haemolymph.	[53]
	*Bombyx mori*	Recombinant 30 K protein	HeLa cells; Spodoptera frugiperda (Sf9) cells	In vitro	In human and insect cells, recombinant 30 K protein prevents apoptosis triggered by viruses or chemicals.	A number of human disorders linked to apoptosis may benefit from the use of recombinant 30 K protein.	[54]
	*Bombyx mori*	Silkworm haemolymph	The vaccinia virus–HeLa cell system	In vitro	Silkworm haemolymph inhibited apoptosis, which reduced cell detachment from an adhering surface.	In commercial animal cell cultures, silkworm pupae haemolymph is helpful in preventing cell death.	[55]
Blood pressure reduction	—	Peptide hydrolysates	Spontaneously hypertensive rats	In vivo	In the treated group, there was a dose-related drop in systolic blood pressure. In normal and non-hypertensive rats, peptide hydrolysate had no effect on systolic blood pressure.	The peptide hydrolysate in silkworm pupae protein possesses antihypertensive effect that is both safe and healthy, which will aid in the investigation of silkworm protein peptides as a functional component of antihypertensive therapy.	[56]
	*Bombyx mori*	Protein hydrolysates	RP- HPLC	In vitro	Angiotensin I-converting enzyme inhibitory action is found in silkworm protein hydrolysates.	Angiotensin I-converting enzyme inhibitor medicines could come from this source.	[57]
	*Bombyx mori*	Protein hydrolysates	RP- HPLC	In vitro	With a half-inhibitory concentration of 102.15 M, the tripeptide inhibited the angiotensin-converting enzyme; the mode of angiotensin-converting enzyme inhibition was competitive.	It can be utilised in antihypertensive supplemental therapy foods as a functional element.	[58]
	*Bombyx mori*	Protein hydrolysates	HPLC	In vitro	By flexible docking calculation, the peptide inhibitory activity was 0.047 mg/mL in IC50, and it was bound to Asp415, Asp453, Thr282, His 353, and Glu162 in the hydrogen bond to the angiotensin-converting enzyme active pocket.	It could be a good idea to look into functional foods that have antihypertension bioactivity.	[59]
Blood lipid reduction; weight loss	*Bombyx mori*	Protein hydrolysates	3T3-L1 cells	In vitro	Upregulation of GLUT4 increases glucose absorption, whereas upregulation of leptin lowers fat storage.	For the first time, silk protein hydrolysate decreased fat accumulation by affecting leptin up-regulation during 3T3-L1 preadipocyte development into fibroblasts.	[60]
	*Bombyx mori*	Peptides	3T3-L1 preadipocytes	In vitro	Adipogenesis is inhibited when adipogenic gene expression and protein synthesis are blocked, resulting in a decrease in body weight gain.	Alternatives to reduce dietary obesity that have no negative effects could be viable options.	[61]
	—	Oil	Sprague– Dawley rats	in vivo	Consumption of silkworm pupae stimulates fat metabolism, lowering blood lipid levels.	The consumption of silkworm pupae reduces fat storage, which is thought to be useful in the prevention of metabolic syndrome.	[62]
Blood glucose regulation	*Bombyx mori*	Soluble fibroin	3T3-L1 adipocyte	in vitro	In 3T3-L1 adipocytes, fibronectin promotes glucose absorption and metabolism.	This could explain why the body’s response to fibre improves diabetic hyperglycaemia.	[63]
	*Bombyx mori*	Purified fibroin	A Spanish hybrid of silkworm races (Sierra Morena X Bagdad)	In vivo	The findings show a decrease in glucose levels in the haemolymph.	Diabetes, obesity, and other lifestyle-related disorders may benefit from this supplement.	[64]
	*Bombyx mori*	Protein	Male C57BL/6 mice	in vivo	The protein from silkworm pupae lowers blood glucose levels considerably.	—	[61]
Cardiovascular protection	*Bombyx mori*	Crude extract	Male New Zealand white rabbits	in vivo	The size of the atherosclerotic plaques was reduced histopathologically.	There is a scientific basis for naming *Bombyx mori* cocoon extract as a cardioprotective and neuroprotective medication.	[65]
	—	Silkworm pupae oil sodium salt	Rat VSMCs cells	In vitro	ERK1/2 phosphorylation was downregulated in PDGF-bb-stimulated VSMCs, which inhibited PDGF-bb-induced cell migration and proliferation.	As a functional food and medicine, it could be beneficial in the treatment of vascular problems.	[66]
	*Bombyx mori*	Silkworm Protein 30Kc6	In vivo atherosclerosis rabbit model	In vivo	It reduced serum levels of total triglycerides (TGs), high-density lipoprotein cholesterol (HDL-C), low-density lipoprotein cholesterol (LDLC), and total cholesterol (TC) in atherosclerotic rabbits, improving their condition.	Providing critical information for the prevention and treatment of human cardiovascular disease.	[52]
Liver protection	*Bombyx mori*	Oil	Acetaminophen-induced acute liver injury Kunming mice model	In vivo	Silkworm pupa oil reduced MDA levels while enhancing SOD and GSH-Px activity, preventing APAP-induced oxidative stress. Overall, silkworm pupa oil protected against APAP-induced liver injury, which was related to the suppression of the oxidative stress-mediated NF-B signalling pathway; SPO also protected against APAP-induced liver injury.	Silkworm pupa oil supplementation could be a viable treatment option for acute liver damage.	[67]
	*Bombyx mori*	Fermented silkworm powder	Sprague–Dawley rats	In vivo	It significantly reduces hepatic alcohol dehydrogenase and acetaldehyde dehydrogenase activity and significantly increases serum AST, g-GTP, and LDH activity, as well as blood alcohol and acetaldehyde levels.	A promising pharmacological candidate for the prevention of hepatotoxicity and oxidative stress caused by alcohol.	[68]
Treatment of gastric ulcers	*Bombyx mori*	Oil	Hydrochloric acid/ethanol-induced gastric ulcers Kunming mice model	In vivo	Gastric ulcer area and secretions were reduced by silkworm pupae oil, but gastric pH increased. SOD, CAT, GSH-Px, SST, and VIP serum levels increased, while IL-6, IL-12, TNF-α, IFN-γ, MTL, and GT serum levels dropped. Meanwhile, EGF, EGFR, VEGF, and eNOS expressions were increased, while NF-κB, Bcl-2, COX-2, and iNOS expressions were decreased.	In mice, silkworm pupa oil decreased oxidative damage and inflammation.	[69]
Immune regulation	*Bombyx mori*	Polysaccharide	Penaeid prawns	In vivo	Innate immunity is turned on.	In penaeid prawns, it effectively inhibits vibriosis.	[41]
	*Bombyx mori*	Peptides	Mouse spleen cells	In vitro	Immune-related factors such as interleukin-6 and interleukin-12, nuclear factor-B, cyclin D1, and cyclin-dependent kinase 4 are all stimulated.	Silkworm pupae polysaccharides have an immunomodulatory function and may have medicinal promise.	[70]
Antigenotoxic	*Antheraea assamensis*	—	Normal human leukocytes; Comet assay	In vitro	At a dosage of 1 mg/mL, pupae extract protects against hydrogen peroxide-induced DNA damage.	The inclusion of polyphenolic groups and fatty acids such as linoleic acid may explain pupae’s antigenotoxic activity.	[35]
As bioreactor	*Bombyx mori*	—	Silkworm pupae	In vivo	Silkworm nucleopolyhedrosis virus was used to successfully express the human granulocyte-macrophage colony-stimulating factor in silkworm pupae.	For heterologous protein expression, the silkworm pupa is a convenient and low-cost bioreactor.	[71]
Antifatigue	*Bombyx mori*	Powders of silkworm, pupae, dongchunghacho, and silk powder	ICR mice	in vivo	Increases swimming time and muscle mass in mice while reducing tiredness.	It has antifatigue properties and may help athletes perform better.	[72]
Anti-Alzheimer’s disease	*Bombyx mori*	Silkworm pupa vaccine	Transgenic mouse model of AD	In vivo	Recombinant proteins synthesised in domestic silkworm pupae reduced brain deposition, lowered malondialdehyde levels, and improved memory and cognitive performance in mice.	The very nutritious CTB-A15 silkworm pupae vaccine could be used to prevent Alzheimer’s disease in the future.	[73]
	*Bombyx mori*	Silkworm pupae Powder	Male Wistar rats		Hippocampal memory deficit was significantly reduced in vivo, as was hippocampal neuron density.	Silkworm pupae appear to be a potentially useful meal for Alzheimer’s disease prevention. Hippocampal memory deficit was significantly reduced, as was hippocampal neuron density.	[74]
Alcohol detoxification	*Bombyx mori*	Extracts	Imprinting Control Region (ICR) mice	In vivo	There is a significant increase in alcohol dehydrogenase activity in the livers of mice given 0.5 mg/mL silkworm pupae extract orally.	The positive effect of silkworm pupae extract on animal alcohol detoxification suggests that the extract could be employed as a therapeutic substance to help people avoid hangovers.	[75]
Antiageing	*Bombyx mori*	Oils and sericin	—		Oils and sericin have tyrosinase inhibitory and free-radical scavenging properties in vitro.	Antiaging and whitening cosmetics are possible applications.	[76]
Inhibits the proliferation of fibroblasts	—	Carboxymethyl chitosan	Mouse L929 fibroblasts	In vitro	SP-carboxymethyl chitosan suppresses cell growth and drastically reduces TGF-β1/Smads signalling pathway gene and protein expression.	Through the TGF-β1/Smads signalling pathway, SP-carboxymethyl chitosan may impact L929 cell proliferation and inhibit postoperative adhesion.	[43]

“—” indicates not stated in the literature.

## Data Availability

The data used in this study are available in this article.
